# Supplementary material for: Acute Kidney Injury and Septic Shock—Defined by Updated Sepsis-3 Criteria in Critically Ill Patients
Source: J Clin Med. 2019 Oct 18;8(10):1731. doi: 10.3390/jcm8101731 (PMC6832288; doi:10.3390/jcm8101731)
Supplement: Supplementary file 1 [file jcm-08-01731-s001.pdf]

Supplemental

Material and methods

Clinical Data Collection

Comorbidities were defined as followed: diabetes mellitus (DM): previous usage of insulin, glucagon-like peptide-1 receptor agonists or oral hypoglycemic agents; hypertension: use of anti-hypertensive agents or blood pressure > 140/90 mmHg at the time of hospitalization; heart failure: New York Heart Association (NYHA) functional class IV; chronic kidney disease (CKD): baseline estimated glomerular filtration rate (eGFR) ≦ 60 mL/min/1.73m^2 for more than 3 months.

Definition of basal kidney function

We defined baseline serum creatinine (sCr) as most recent inpatient or outpatient value as baseline the value obtained 1. the value obtained pre-admission stable outpatient measurements 2. at hospital discharge at the previous admission in those who had more than one admission, or 3. the value estimated using the Modification of Diet in Renal Disease equation in those without previous records (assuming an average eGFR of 75 ml/min/1.73m^2) [1, 2]. The peak sCr was defined as the highest sCr before RRT initiation in ICUs.

Dialysis setting

The RRT modalities for individual patients were chosen by the clinical team and occasionally changed later according to patients' hemodynamics. For those who needed cardiovascular support with catecholamines of more than 15 mcg/kg/min to maintain systemic blood pressure up to 120 mmHg, continuous veno-venous hemofiltration (CVVH) was prescribed. The hemofiltration flow and blood flow were 25ml/kg/hour and 200ml/min, respectively. Replacement fluid was bicarbonate-buffered and was pre-dilutionally administered at a dynamically adjusted rate to achieve the desired fluid therapy goals. For patients with required IE of 5-15 mcg/kg/min, sustained low efficiency daily dialysis (SLEDD) or diafiltration (SLEDD-f) was used with blood flow of 200ml/min, dialysate flow of 300ml/min, and hemofiltration flow of 25ml/kg/hour. The duration was around 6-12 hrs depending on the amount of ultrafiltration required. Intermittent hemodialysis was performed for 4 hours except for the first and second sessions. We used low-flux polysulfone hemofilters (KF-18C, Kawasumi Laboratories, Japan) with a dialysate and blood flow of 500ml/min. [3-6] Double lumen catheters were placed for vascular access.

Supplementary Statistics

We generated receiver-operating characteristics (ROC) curves and calculated the area under the curve (AUC) to measure the performance of candidate criteria. Multiple comparisons were analyzed using one-way analysis of variance (ANOVA).

1. Generalized additive model (GAM)

Agreement between the change of SOFA score, and qSOFA, were determined using Cronbach α with 95% CIs generated with boot strapping (500 samples).

In order to reveal the effects of delta SOFA on mortality for an individual patient, a generalized additive model (GAM) (with spline) incorporating the subject-specific (longitudinal) random effects was plotted with adjustment for other clinical parameters to predict the outcomes [7, 8]. We defined the optimal cut-off value as when the log odd equals to zero[9].

2. Assessing the performance of prediction models, decision curve analysis (DCA)

Clinical usefulness and net benefit of the Sepsis-3 scores were estimated according with decision curve analyses (DCA)[10], in order to identify patients who had any of the adverse events of interest. The DCA

shows the clinical usefulness of each new model based on a continuum of potential thresholds for adverse events (x-axis) and the net benefit of using the model to stratify patients at risk (y-axis) relative to assuming that no patient will have an adverse event. The basic interpretation of DCA is that the strategy with the highest net benefit at a particular threshold probability has the highest clinical value. In this study, the prediction models are represented by dashed lines (original scores) and solid lines (modified scores with Sepsis3 and AKI risk prediction score). Those models that are the farthest away from the horizontal black line (i.e., assuming no adverse event) demonstrate the higher net clinical benefit.

3. Increased SOFA and 90-day mortality (figure S3)

A GAM plot was generated and showed a positive correlation between increased SOFA at dialysis initiation and ICU admission and the log of the odds of the 90-day mortality after adjusting for nonlinear effects of the variables listed in Table 1.

Accumulated hazard plots were developed by Cox regression models for the outcome of interest. In view of the high mortality rate among dialysis patients, competing-risk regression analysis based on the Fine and Gray model was performed using cumulative incidence and sub-distribution hazard functions[11].

All analyses were performed with R software, version 3.2.2 (Free Software Foundation, Inc., Boston, MA), MedCalc Statistical Software, version 15.11.3 (MedCalc Software bvba, Ostend, Belgium; https://www.medcalc.org; 2015) and Stata/MP version 12 (Stata Corporation, TX) for competing-risk analysis. A p-value <0.05 was considered significant.

Ethics

The CAKS study was approved by the institutional review boards of the participating institutions. The need for informed consent was waived because all personal data was fully de-identified and only data that were routinely collected for clinical purposes were analysed. (Approval No. NRPB2014050014).

Supplementary results

Sepsis perSepsis-3 criteria versus 90-day outcomes

The Sepsis-3 criteria in its totality were more predictive of 90 day mortality than the individual components. ROC curve analysis showed an AUC of 0.631 for qSOFA [95% Confidence interval (CI) 0.601-0.660] for prediction of 90-day mortality. The AUC was 0.520 for delta SOFA ≥2 (95% CI, 0.490-0.550) and 0.650 for the entire Sepsis-3 definition (95% CI 0.621-0.679) (Sepsis-3 versus qSOFA, p=0.004; Sepsis-3 versus delta SOFA ≥2, p≤0.001). (Figure s2a, Table S2) The prognostic performance of the Sepsis-3 criteria was similar to the AKI risk prediction score (AUC 0.688, p=0.063). The Sepsis-3 criteria had the best results for prediction of the composite outcome of mortality or dialysis dependence at 90 days. (Figure s2b)

The Sepsis-3 criteria performed significantly better at prognosticating mortality (area under the Receiver Operating Characteristics curve 0.650) than delta SOFA ≥2 (0.520) alone or qSOFA ≥2 (0.631) alone. The Sepsis-3 criteria had a greater outcome association than the delta SOFA or qSOFA ≥2 in isolation or the AKI risk score.

In addition, a significantly greater proportion of survivors were treated in metropolitan hospitals compared to medical centers. On the day when RRT was started, serum lactate concentrations were higher in those who subsequently died. Mechanical ventilation and need for catecholamine support were also more common in non-survivors.

The proportion of patients with liver cirrhosis or cancer was also higher. At initiation of RRT, patients with sepsis or septic shock had significantly higher severity of illness scores and higher serum lactate results and required mechanical ventilation and catecholamine treatment more often. Hospital mortality, 90-day mortality and risk of dialysis dependence at 90 days were highest in those with septic shock. (figure S3,4,5)

Supplementary discussion

The qSOFA criteria were not intended to replace SIRS in the definition of sepsis, but instead aim to facilitate the identification of early sepsis outside the ICU[12]. Our results suggest that qSOFA score 2 or higher had similar predictive power as the Sepsis-3 criteria in their totality in predicting 90-day mortality. In fact, since the qSOFA score does not include any creatinine results, it may be particularly useful for patients with AKI-D. Of note, about 90% of AKI-D patients with a positive qSOFA also met the Sepsis-3 criteria. Although the discrimination ability of both Sepsis-3 criteria and qSOFA was not perfect (a finding that has also been shown in the general population, [13, 14]), we found that both were able to identify a high-risk cohort with a particularly high risk of mortality. (Table S2)

These examples illustrate the need for relatively simple criteria that have been validated in the patient cohort of interest and have been accepted by clinicians and researchers. Our results provide some assurance that the Sepsis-3 criteria are applicable to AKI-D patients and indeed identify those with a high risk of important outcomes.

Using the GAM model adjusted by the disease severity, we could define the best cut-off values of change of SOFA from baseline by more than 10 showing that qSOFA levels at dialysis initializing could predict risk of mortality. The best cut-off points predicting 90 day mortality were a change of SOFA score by more than 10.

Conflicts of Interest Statement: The authors report no potential conflicts of interest relevant to this article.

Table S1. Integer risk score for prediction of 60-day mortality in critically ill patients with AKI requiring dialysis.

**Parameters**	**Score Points**							
	**0**	**1**	**2**	**3**	**4**	**5**	**12**	**14**
Chronic health condition								
age	<=50	51-56	57-62	63-68	69-74	>74		
chronic hypoxemia	No			Yes				
cardiovascular disease	No	Yes						
malignancy	No					Yes		
immunosuppressive therapy	Yes		No					
Present illness								
ischemic AKI	No	Yes						
postsurgery	Yes		No					
At RRT initiation								
heart rate (beats/min)	<=96	97-126	>126					
mean arterial pressure (mmHg)	>=86	73-85	61-72	<61				
urine volume (ml/day)	<=501	151-500	41-150	<41				
Mechanical ventilation and FiO2:								
mechanical ventilation and FiO2< 0.60	No					Yes		
no Mechanical ventilation and FiO2 >=0.60	No							Yes
mechanical ventilation and FiO2 >=0.60	No						Yes	
arterial Ph	>7.3	7.2-7.3	<7.2					
arterial oxygen partial pressure (mmHg)	>96	<=96						
serum creatinine (mg/dl)	>4.7	3.7-4.7	<3.7					
serum bicarbonate (mmol/L)	>25	18-25	<18					
serum phosphate (mg/dl)	<=3	>3						
serum albumin (g/dl)	>=2.5	1.9-2.4	<1.9					
total bilirubin (mg/dl)	<=1	1.1-1.9	2-3.7	>3.7				
INR	<=1.2	1.3-1.8	>1.8					
platelet count (k/ l)	>=165	91-165	50-90	<50				

AKI, acute kidney injury; FiO2, fraction of inspired oxygen; INR, international normalized ratio; RRT, renal replacement therapy.
aRisk score minimum (0), maximum (59); total score is calculated as the arithmetic sum of the points for the individual parameters.

Abbreviations; AKI, acute kidney injury; FiO2, fraction of inspired oxygen; INR, international normalized ratio; RRT, renal replacement therapy. aRisk score minimum (0), maximum (59); total score is calculated as the arithmetic sum of the points for the individual parameters. This AKI risk prediction score was proposed by Demirjian et al.[15].

Table S2. Diagnostic performance of Sepsis-3 criteria in prediction of 90-day hospital mortality.

	**qSOFA>=2**	**Δ SOFA>=2**	**Septic shock**
Sensitivity, (95%CI)	0.638 (0.601 - 0.675)	0.991 (0.981 - 0.997)	0.632 (0.595 - 0.669)
Specificity, (95%CI)	0.623 (0.574 - 0.670)	0.049 (0.030 - 0.075)	0.626 (0.577 - 0.673)
Predictive value, (95%CI)			
Positive	0.737 (0.699 - 0.772)	0.633 (0.603 - 0.662)	0.737 (0.699 - 0.772)
Negative	0.510 (0.465 - 0.555)	0.769 (0.564 - 0.910)	0.507 (0.462 - 0.552)
Likelihood ratio, (95%CI)			
Positive	1.694 (1.477 - 1.944)	1.042 (1.018 - 1.067)	1.689 (1.471 - 1.940)
Negative	0.580 (0.512 - 0.658)	0.181 (0.073 - 0.448)	0.588 (0.519 - 0.665)
AUROC, (95% CI)	0.631 (0.601 - 0.661)	0.520 (0.509 - 0.531)	0.629 (0.599 - 0.659)

Abbreviations: AUROC, area under the receiver operating characteristic curve; CI, confidence interval; qSOFA, quick Sequential Organ Failure Assessment; Δ , delta; .

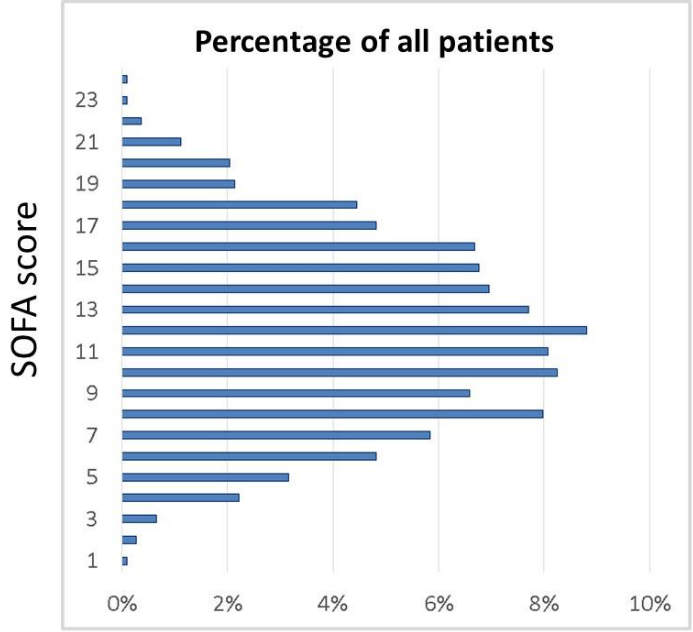

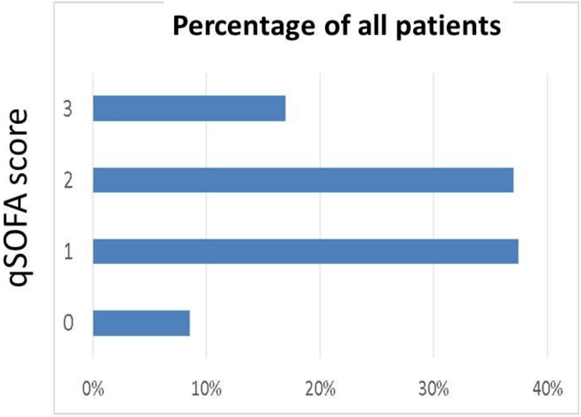

Figure S1. Distribution of Patients by SOFA Score, and qSOFA Score at initiation of acute dialysis (N = 1,078), X axis is %. **Abbreviations**: qSOFA, quick Sequential Organ Function Assessment; SOFA, Sequential Organ Function Assessment.

Figure S2. Prognostic Accuracy of Sepsis-3 components among AKI-D patients with suspected or confirmed infection at dialysis initiation. a) 90 day mortality Receiver-operator characteristic curves discriminate (denoted area under the receiver operating characteristic curve): Sepsis-3, (0.650), qSOFA score ≥2 (0.631), AKI risk prediction score (0.688) and increased SOFA ≥2 (0.520). b) 90 day composite outcomeReceiver-operator characteristic curves discriminate (denoted area under the receiver operating characteristic curve): Sepsis-3, (0.587), qSOFA score ≥2 (0.578), AKI risk prediction score (0.596) and increased SOFA ≥2 (0.501). **Abbreviation**: AKI, acute kidney injury; SOFA, Sequential Organ Failure Assessment; aSOFA, quick Sequential Organ Failure Assessment;

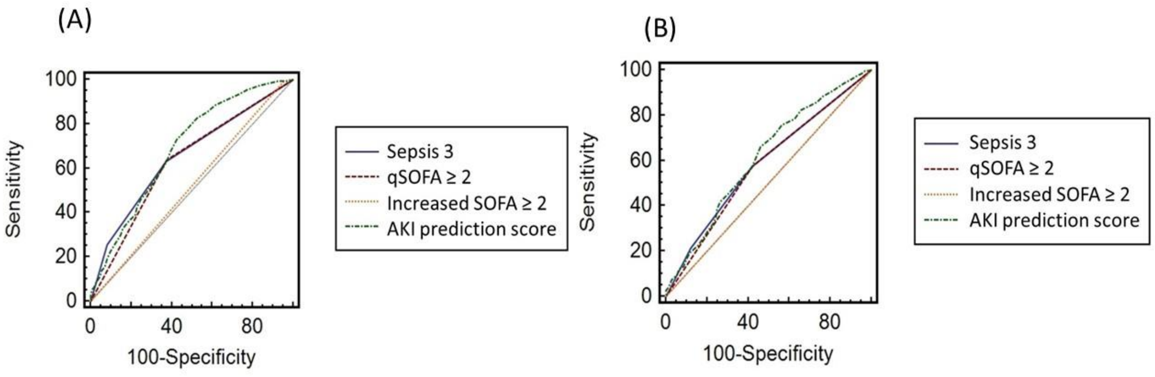

Figure S2. Prognostic Accuracy of Sepsis-3 components among AKI-D patients with suspected or confirmed infection at dialysis initiation. a) 90 day mortality Receiver-operator characteristic curves discriminate (denoted area under the receiver operating characteristic curve): Sepsis-3, (0.650), qSOFA score ≥2 (0.631), AKI risk prediction score (0.688) and increased SOFA ≥2 (0.520). b) 90 day composite outcomeReceiver-operator characteristic curves discriminate (denoted area under the receiver operating characteristic curve): Sepsis-3, (0.587), qSOFA score ≥2 (0.578), AKI risk prediction score (0.596) and increased SOFA ≥2 (0.501). **Abbreviation**: AKI, acute kidney injury; SOFA, Sequential Organ Failure Assessment; aSOFA, quick Sequential Organ Failure Assessment;

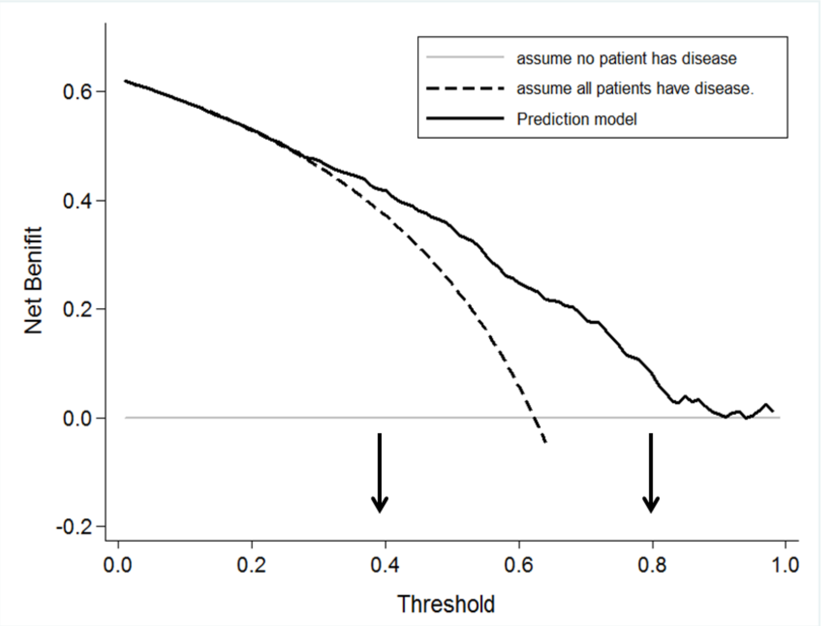

Figure S3. Decision curve analysis (DCA) plot to assess the clinical consequences of screening AKI-D patients for risk of 90 day mortality using sepsis-3 score in addition to AKI risk prediction score[16]**.** Y-axis is the net benefit of the decision strategy. Net benefit is the net proportion of patients with 90 day mortality in whom a prediction model would provide benefit without applying a prediction model to patients with good outcomes. For AKI patients initiated on dialysis, forecasting with the AKI predicting model and Sepsis-3 criteria in combination would yield no net benefit. For risk thresholds between 30 and 80% the superior strategy, forecasting with the AKI risk prediction score and Sepsis-3 is beneficial. For moderate to high-risk thresholds (80 to 100%), there is no net benefit from using the AKI risk prediction score together with the Sepsis-3 model.

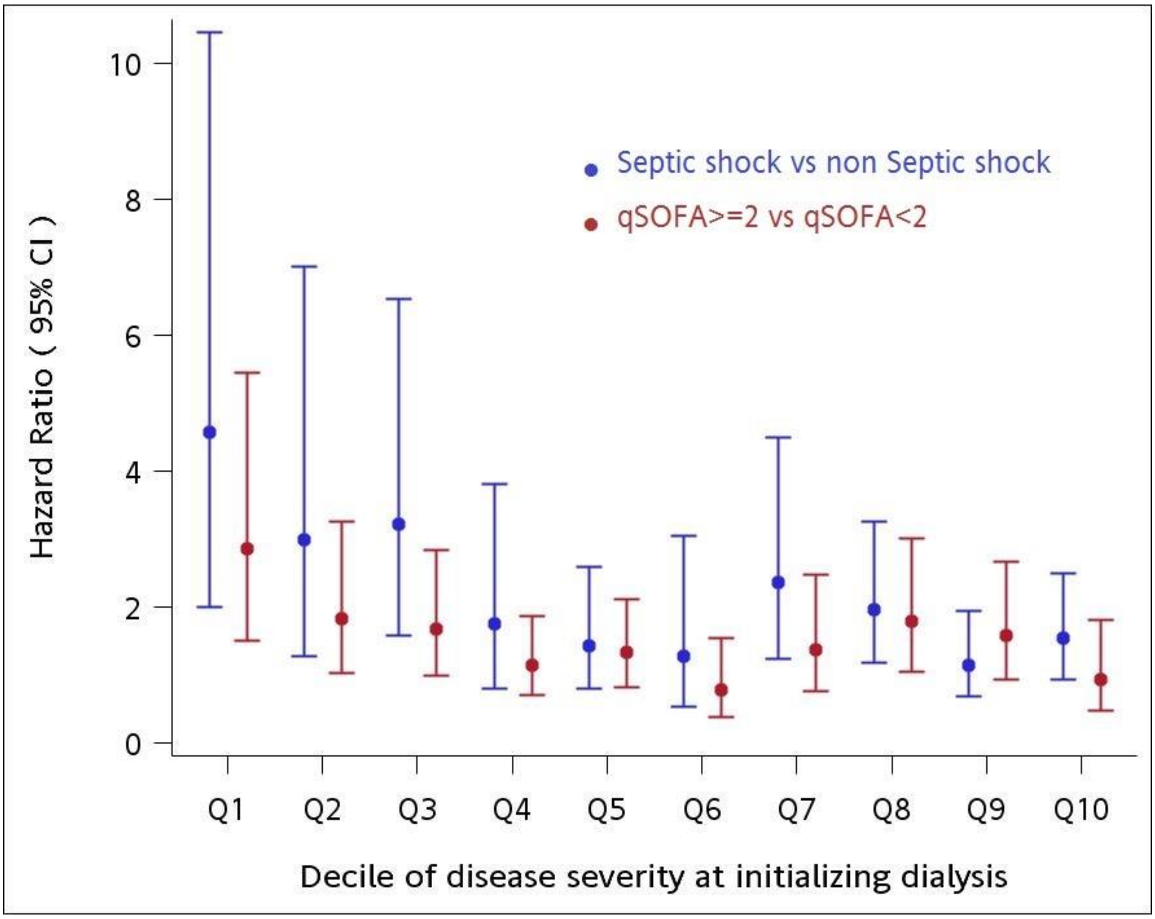

Figure S4. Hazard Ratios for 90-day Mortality comparing different criteria of Sepsis-3 definition. Abbreviations: APACHE, Acute Physiology and Chronic Health Evaluation score; AKI-D, acute kidney injury with dialysis; CI, confidence interval; qSOFA, quick Sequential Organ Failure Assessment, Comparison between different categories of sepsis for each decile of baseline risk according to APACHE II score in AKI-D patients with suspected infection (N = 1078). Error bars indicate the 99% Confidence Intervals. The x-axis divides the cohort into deciles of APACHE II score at initiation of dialysis.

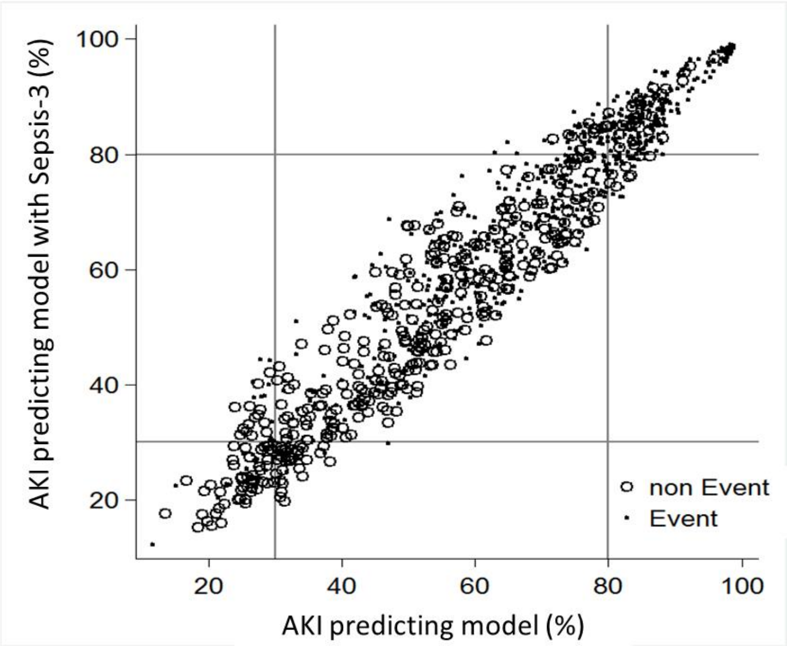

Figure S5. Scatter plot of AKI risk prediction forecasted probabilities without and with the Sepsis-3 score. Note that some AKI-D patients have higher predicted risks in the model with Sepsis- 3 values than in the model without Sepsis-3 (dots in right lower corner of the graph).

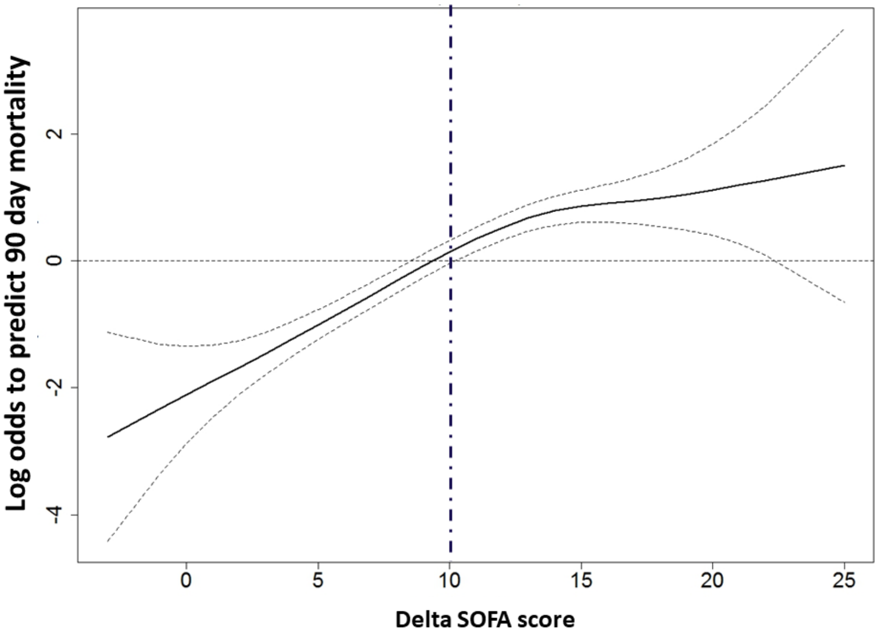

Figure S6. Generalized additive model (GAM) plot for the probability of 90-day mortality against delta SOFA, in term of the difference of SOFA at initializing dialysis and ICU admission, initiating the subject-specific (longitudinal) random effects expressed as the logarithm of the odds (logit). The relationship of delta SOFA with these variables was further illustrated by GAM analysis, adjusted for Acute Physiology and Chronic Health Evaluation (APACHEII) at ICU admission, sex and age, showing that qSOFA levels at dialysis initializing could predict risk of mortality. GAM results showed the best cut-off points predicting 90 day mortality were a change of SOFA score by more than 10. Abbreviations; SOFA, sequential organ failure assessment;.

References

1. Wu VC, Huang TM, Lai CF, Shiao CC, Lin YF, Chu TS, Wu PC, Chao CT, Wang JY, Kao TW *et al*: **Acute-on-chronic kidney injury at hospital discharge is associated with long-term dialysis and mortality**. *Kidney Int* 2011, **80**(11):1222-1230.
2. Siew ED, Ikizler TA, Matheny ME, Shi Y, Schildcrout JS, Danciu I, Dwyer JP, Srichai M, Hung AM, Smith JP *et al*: **Estimating baseline kidney function in hospitalized patients with impaired kidney function**. *Clin J Am Soc Nephrol* 2012, **7**(5):712-719.
3. Wu VC, Ko WJ, Chang HW, Chen YW, Lin YF, Shiao CC, Chen YM, Chen YS, Tsai PR, Hu FC *et al*: **Risk factors of early redialysis after weaning from postoperative acute renal replacement therapy**. *Intensive Care Med* 2008, **34**(1):101-108.
4. Wu VC, Ko WJ, Chang HW, Chen YS, Chen YW, Chen YM, Hu FC, Lin YH, Tsai PR, Wu KD: Early renal replacement therapy in patients with postoperative acute liver failure associated with acute renal failure: effect on postoperative outcomes. *Journal of the American College of Surgeons* 2007, 205(2):266-276.
5. Shiao CC, Wu VC, Li WY, Lin YF, Hu FC, Young GH, Kuo CC, Kao TW, Huang DM, Chen YM *et al*: Late initiation of renal replacement therapy is associated with worse outcomes in acute kidney injury after major abdominal surgery. *Critical care (London, England)* 2009, 13(5):R171.
6. Shiao CC, Ko WJ, Wu VC, Huang TM, Lai CF, Lin YF, Chao CT, Chu TS, Tsai HB, Wu PC *et al*: U-curve association between timing of renal replacement therapy initiation and in-hospital mortality in postoperative acute kidney injury. *PloS one* 2012, 7(8):e42952.
7. Wu VC, Lo SC, Chen YL, Huang PH, Tsai CT, Liang CJ, Kuo CC, Kuo YS, Lee BC, Wu EL *et al*: **Endothelial progenitor cells in primary aldosteronism: a biomarker of severity for aldosterone vasculopathy and prognosis**. *J Clin Endocrinol Metab* 2011, **96**(10):3175-3183.
8. Wu VC, Lai CF, Shiao CC, Lin YF, Wu PC, Chao CT, Hu FC, Huang TM, Yeh YC, Tsai IJ *et al*: **Effect of diuretic use on 30-day postdialysis mortality in critically ill patients receiving acute dialysis**. *PloS one* 2012, **7**(3):e30836.
9. Hin LY, Lau TK, Rogers MS, Chang AM: Dichotomization of continuous measurements using generalized additive modelling--application in predicting intrapartum caesarean delivery. *Stat Med* 1999, 18(9):1101-1110.
10. Vickers AJ, Cronin AM, Elkin EB, Gonen M: Extensions to decision curve analysis, a novel method for evaluating diagnostic tests, prediction models and molecular markers. *BMC Med Inform Decis Mak* 2008, 8:53.
11. Dignam JJ, Zhang Q, Kocherginsky M: **The use and interpretation of competing risks regression models**. *Clin Cancer Res* 2012, **18**(8):2301-2308.
12. Vincent JL, Martin GS, Levy MM: **qSOFA does not replace SIRS in the definition of sepsis**. *Critical care (London, England)* 2016, **20**(1):210.
13. Seymour CW, Liu VX, Iwashyna TJ, Brunkhorst FM, Rea TD, Scherag A, Rubenfeld G, Kahn JM, Shankar-Hari M, Singer M *et al*: Assessment of Clinical Criteria for Sepsis: For the Third International Consensus Definitions for Sepsis and Septic Shock (Sepsis-3). *JAMA* 2016, 315(8):762-774.
14. Raith EP, Udy AA, Bailey M, McGloughlin S, MacIsaac C, Bellomo R, Pilcher DV, Australian, New Zealand Intensive Care Society Centre for O, Resource E: **Prognostic Accuracy of the SOFA Score, SIRS Criteria, and qSOFA Score for In-Hospital Mortality Among Adults With Suspected Infection Admitted to the Intensive Care Unit**. *JAMA* 2017, **317**(3):290-300.
15. Demirjian S, Chertow GM, Zhang JH, O'Connor TZ, Vitale J, Paganini EP, Palevsky PM, Network VNARFT: **Model to predict mortality in critically ill adults with acute kidney injury**. *Clin J Am Soc Nephrol* 2011, **6**(9):2114-2120.
16. Parving HH, Brenner BM, McMurray JJ, de Zeeuw D, Haffner SM, Solomon SD, Chaturvedi N, Persson F, Desai AS, Nicolaides M *et al*: **Cardiorenal end points in a trial of aliskiren for type 2 diabetes**. *N Engl J Med* 2012, **367**(23):2204-2213.
